# Anti‐inflammatory and immune‐modulatory impacts of berberine on activation of autoreactive T cells in autoimmune inflammation

**DOI:** 10.1111/jcmm.16049

**Published:** 2020-11-01

**Authors:** Seyed‐Morteza Ehteshamfar, Masoume Akhbari, Jalil Tavakol Afshari, Motahareh Seyedi, Banafsheh Nikfar, Abbas Shapouri‐Moghaddam, Erfan Ghanbarzadeh, Amir Abbas Momtazi‐Borojeni

**Affiliations:** ^1^ Department of Immunology Faculty of Medicine BuAli Research Institute Mashhad University of Medical Sciences Mashhad Iran; ^2^ Department of Molecular Medicine School of Medicine Qazvin University of Medical Sciences Qazvin Iran; ^3^ Department of Biology Faculty of Science Yazd University Yazd Iran; ^4^ Pars Advanced and Minimally Invasive Medical Manners Research Center Pars Hospital Iran University of Medical Sciences Tehran Iran; ^5^ School of Medicine Gilan University of Medical Sciences Gilan Iran; ^6^ Department of Medical Biotechnology Faculty of Medicine Mashhad University of Medical Sciences Mashhad Iran

**Keywords:** autoimmunity, berberine, dendritic cell, inflammation, macrophage, T helper cell

## Abstract

Autoreactive inflammatory CD4^+^ T cells, such as T helper (Th)1 and Th17 subtypes, have been found to associate with the pathogenesis of autoimmune disorders. On the other hand, CD4^+^ Foxp3^+^ T regulatory (Treg) cells are crucial for the immune tolerance and have a critical role in the suppression of the excessive immune and inflammatory response promoted by these Th cells. In contrast, dendritic cells (DCs) and macrophages are immune cells that through their inflammatory functions promote autoreactive T‐cell responses in autoimmune conditions. In recent years, there has been increasing attention to exploring effective immunomodulatory or anti‐inflammatory agents from the herbal collection of traditional medicine. Berberine, an isoquinoline alkaloid, is one of the main active ingredients extracted from medicinal herbs and has been shown to exert various biological and pharmacological effects that are suggested to be mainly attributed to its anti‐inflammatory and immunomodulatory properties. Several lines of experimental study have recently investigated the therapeutic potential of berberine for treating autoimmune conditions in animal models of human autoimmune diseases. Here, we aimed to seek mechanisms underlying immunomodulatory and anti‐inflammatory effects of berberine on autoreactive inflammatory responses in autoimmune conditions. Reported data reveal that berberine can directly suppress functions and differentiation of pro‐inflammatory Th1 and Th17 cells, and indirectly decrease Th cell‐mediated inflammation through modulating or suppressing other cells assisting autoreactive inflammation, such as Tregs, DCs and macrophages.

## 1 INTRODUCTION

### 1.1 Autoreactive Th1 and T17 cells

Autoreactive CD4^+^ T cells, such as T helper (Th)1 and Th17 subtypes, have been found to involve in the pathogenesis of several autoimmune disorders, including multiple sclerosis (MS), inflammatory bowel disease (IBD) and rheumatoid arthritis (RA). Th1 cells, which predominantly produce interferon gamma (IFNγ), participate in the elimination of intracellular pathogens and are involved in cell‐mediated and delayed‐type hypersensitivity responses. There are several lines of evidence that Th1 cells producing IFNγ are closely correlated with the clinical severity of autoimmune diseases and can independently transfer diseases into naïve mice. In mice with experimental autoimmune encephalomyelitis (EAE) as a model of human MS, IFNγ levels within the central nervous system (CNS) have been found to associate with disease severity, with high levels detected at the peak of disease that fall as the disease spontaneously subsides into remission.^1^, ^2^ Infiltrating CD4^+^ T cells were found as the source of this IFNγ,^3^ and the adoptive transfer of IFNγ‐producing T‐cell lines has been demonstrated to promote autoimmune pathologies.^2^, ^4^ The role of Th1 in autoimmune disorders is further confirmed by findings that mice lacking the Th1 lineage‐specific transcription factors, T‐bet and signal transducer and activator of transcription 4 (STAT4), are protected from the disease.^5^ The pathogenic role of Th1 cells has been also declared in other models of autoimmunity, such as adjuvant‐induced arthritis (AIA) as a model of human RA,^6^ experimental autoimmune uveitis (EAU)^7^, ^8^ and experimental autoimmune myocarditis (EAM).^9^, ^10^

In parallel to Th1, some reports show that Th17 cells, a subtype of interleukin (IL)‐17 secreting CD4^+^ Th cells, and their relevant cytokines play important role in the severity and progression of several autoimmune diseases. The pathogenic role of Th17 cells in autoimmune diseases has emerged from studies that indicate IL‐17 expression is elevated at the inflamed sites in patients with RA, MS, uveitis, and psoriasis.^11^, ^12^, ^13^, ^14^, ^15^, ^16^ IL‐17 is a pro‐inflammatory cytokine that affects various cell types, including endothelial cells, fibroblasts, keratinocytes, epithelial cells, and macrophages, and promotes the generation of several cytokines including IL‐6, IL‐1, tumour necrosis factor alpha (TNF‐α), transforming growth factor beta (TGF‐β), granulocyte colony‐stimulating factor (G‐CSF) and granulocyte‐macrophage CSF (GM‐CSF), and many chemokines such as macrophage inflammatory protein 2 (MIP‐2), Cytokine‐induced neutrophil chemoattractant (CINC) and monocyte chemoattractant protein 1 (MCP‐1), as well as prostaglandins like PGE2.^17^, ^18^, ^19^, ^20^, ^21^ A crucial result of such effects is the promotion and recruitment of neutrophils to the inflamed sites.^18^, ^22^ Moreover, IL‐17 is found to induce the generation of matrix metalloproteinases (MMPs) that act to degrade target tissue during the inflammation.^18^ In addition to IL‐17, Th17 cells secret IL‐6, TNF‐α, IL‐21 and IL‐22 cytokines that are attributed to the destructive pro‐inflammatory function of these cells.^18^, ^23^, ^24^ Of note, the impact of the Th17 response on autoimmunity has been evaluated within the various experimental models. Th17/IL‐17 deficient mice were found to has low sensitivity to AIA and EAE,^25^ and treatment with IL‐17R antagonist or IL‐17 neutralizing antibody ameliorated the severity of AIA, EAE and EAU.^22^ It is further supported by findings that show when Th17 regulatory factors, such as IL‐6 and retinoic acid‐related orphan receptor gamma t (RORγt), were knocked out in experimental animals, tissue infiltrating Th17 cells were significantly decreased and autoimmune inflammation was attenuated.^26^

On the other hand, CD4^+^ Foxp3^+^ T regulatory (Treg) cells are crucial for immune tolerance and have a critical role in the suppression of the excessive immune and inflammatory response promoted by autoreactive Th cells.^27^, ^28^ Foxp3^+^ Treg cells can inhibit Th1 and Th17 differentiation and function. The nuclear transcription factor Foxp3, known as a specific marker for Treg cells, plays an essential role in the development and function of Treg cells,^29^ which can suppress differentiation of Th1/Th17 cells by antagonizing the function of the transcription factors RORγt and ROR.^30^ However, IL‐6 overcame this suppressive effect of Foxp3 on Th17 differentiation.^30^ Excessive IL‐6 induces Th17, but suppresses differentiation of Treg cells, shifting the balance of Tregs towards inflammatory Th17 cells in patients with autoimmune disorders. Treg/Th17 imbalance and diminished numbers of Foxp3^+^ Treg cells in patients with various autoimmune diseases are associated with disease severity and activity.^31^ On the contrary, DCs and macrophages, as discussed in the following sections, are immune cells that through their inflammatory functions promote autoreactive T‐cell responses in autoimmune conditions.^32^, ^33^

### 1.2 Berberine: a natural compound possessing immunomodulatory and anti‐inflammatory properties

In recent years, there has been increasing attention to exploring effective immunomodulatory or anti‐inflammatory agents from the herbal collection of traditional medicine. Herbal medicines introduce a rich source of natural compounds for the identification of new therapeutic agents having novel mechanisms of action and for providing valuable insight into new targets involved in the inflammatory process. Among medicinal plants and herbs, numerous plants of the genera Berberis and Coptis have been widely employed in traditional medicine to treat patients with abdominal pain, diarrhoea or gastroenteritis.^34^ Berberine, an isoquinoline alkaloid, is one of the main bioactive ingredients in these herbs (Table 1) and has been shown to exhibit anti‐inflammatory, anti‐oxidation, anti‐atherosclerotic, antimicrobial, antidiabetic, antitumour and neuroprotective effects.^35^, ^36^, ^37^, ^38^, ^39^, ^40^, ^41^, ^42^ Such pleiotropic biological and pharmacological properties of berberine have been suggested to be mainly attributed to its anti‐inflammatory and immunomodulatory properties.^35^, ^43^ Berberine has been found to modulate and/or suppress inflammation through suppressing the production of TNF‐α, IL‐6 and MCP‐1, down‐regulating the expression of cyclooxygenase‐2 (COX‐2), reducing generation of PGE2 and formation of exudates, and inhibiting the expression of MMP‐2 and MMP‐9 through nuclear factor‐kB (NF‐kB) and mitogen‐activated protein kinase (MAPK) signalling cascades.^35^, ^44^, ^45^, ^46^

**TABLE 1:** Various sources of berberine

Family	Scientific name	Common name	Tissue source	Reference
Berberidaceae	Berberis amurensis Rupr.	Barberry	Stem and roots	154
	Berberis concinna Hook.f.		Stem bark	
	Berberis aquifolium Pursh	Oregon grape	Roots	155
	Berberis aristata DC.	Tree turmeric	Bark, roots, raw herb, fruit, stem	156, 157
	Berberis asiatica Roxb. ex DC.	Chutro, rasanjan, marpyashi, daruharidra, darbi	Roots, stem, bark	159, 163, 164
	Berberis beaniana C. K. Schneid.	Kang song xiao bo	—	166
	Berberis aetnensis C.Presl	—	Leaves and roots	167, 168
	Berberis chitria Buch.‐Ham. ex Lindl.	Chitra and indian barberry	Whole plant and roots	170, 171
	Berberis congestiflora Gay	Michay	Leaves and stem	133
	Berberis croatica Mart. ex Schult. & Schult.f.	Croatian barberry	Roots	173
	Berberis floribunda Wall. ex G.Don	Nepal barberry	Roots	174
	Berberis fortunei Lindl.	Fortune's Mahonia	Wood	164
	Berberis japonica R.Br	Japanese Mahonia	Wood, root	164
	Berberis koreana Palib.	Korean barberry	Bark of the stem and roots, seeds, stem, roots	175
	Berberis lycium Royle	Boxthorn barberry	Roots	165
	Berberis microphylla G. Forst.	Patagonian barberry, magellan barberry, calafate	Roots	176
	Berberis umbellata Wall. ex G.Don	Himalayan barberry	Roots	177
	Berberis vulgaris L.	Barberry	Stems and roots	178
Annonaceae	Annickia chlorantha (Oliv.)	African whitewood	Bark	179, 180
	Annickia polycarpa (DC.)	African yellow wood	Bark	180
	Rollinia mucosa (Jacq.) Baill.	Biriba, wild sweet sop, wild cashina	Fruit	181
	Xylopia macrocarpa A.Chev.	Jangkang	Stem bark	164
	Xylopia polycarpa (DC.) Oliv.	—	Stem bark	164
Papaveraceae	Argemone albiflora Hornem	White prickly poppy, Bluestem pricklypoppy	Aerial part and roots	182
	Argemone mexicana L.	Prickly poppy	Apigeal parts, seeds, leaves, roots	183, 184
	Argemone ochroleuca Sweet	Chicalote	Seeds	188
	Argemone platyceras L.	Chicalote poppy, crested poppy	Leaves and stem	189
	Argemone squarrosa Greene	Hedgehog pricklypoppy	Aerial part	190
	Bocconia frutescens L.	Plume poppy, tree poppy, tree celandine, parrotweed, sea oxeye daisy, john crow bush	Leaves, roots, stalks	183, 191
	Chelidonium majus L.	Celandine poppy	Roots	192
	Corydalis chaerophylla DC.	Fitweed	Roots	193
	Corydalis solida subsp brachyloba	Fitweed	Aerial parts	194, 195
	Glaucium corniculatum (L.) Rud. subsp corniculatum	Blackspot Hornpoppy	Aerial parts	196
	Macleaya microcarpa (Maxim.) Fedde	Poppy	Roots	197
Ranunculaceae	Coptis chinensis Franch.	Chinese goldthread	Roots	175
	Coptis japonica (Thunb.) Makino	Japanese goldthread	Rhizome	198
	Coptis teeta Wall.	Gold thread	Rhizome and roots	175, 199
	Hydrastis canadensis L.	Goldenseal	—	200
	Xanthorhiza simplicissima Marshall	Yellowroot	Root, stem, leaves	201
Rutaceae	Phellodendron amurense Rupr.	Amur cork tree	Bark, root bark, trunk bark, perennial Branch bark, annual branches, leaves	199, 202
	Phellodendron chinense C. K. Schneid	Chinese cork tree	Bark	203
	Phellodendron chinense var. glabriusculum C. K. Schneid.	Chinese cork tree	Bark, branch, leaf, bark, heartwood	175, 204, 205
	Phellodendron lavallei Dode	Lavalle corktree	Bark	206
	Zanthoxylum monophyllum (Lam.) P. Wilson	Palo rubio	Stem and branches	207

Several lines of experimental study have recently investigated the therapeutic potential of berberine for treating autoimmune conditions in animal models of MS, RA, IBD and autoimmune uveitis. In the present review article, we seek mechanisms underlying immunomodulatory and anti‐inflammatory effects of berberine on autoreactive inflammatory responses. Based on the reported information, we discuss direct and indirect effects of berberine on autoreactive Th1 and T17 cells, which indirect effects cover pathways by which berberine through affecting Treg cells, DCs and macrophages could suppress inflammatory responses of Th1/Th17 cells in vitro and in vivo in various experimental models of autoimmune diseases (Table 2).

**TABLE 2:** Effects of berberine on cytokine production in various autoimmune diseases

Cytokine	Source of cytokine	Changes of mRNA/protein expression	Effect information	Ref.
*Colitis*				
IL‐1β	‐ Colon tissue ‐ Macrophage ‐ Serum	Decreased protein expression	‐ Reducing inflammatory responses ‐ Inhibiting Th1/Th17 differentiation ‐ Adjusting the M2/M1 ratio	73
IL‐6	‐ Colon tissue ‐ Macrophage ‐ Serum	Decreased protein expression	‐ Reducing inflammatory responses ‐ Inhibiting Th1/Th17 differentiation ‐ Adjusting the M2/M1 ratio	73
IL‐17	‐ T cells of colon tissue ‐ Serum	Decreased mRNA and protein expression	‐ Reducing inflammatory responses ‐ Inhibiting Th1/Th17 differentiation ‐ Improving Treg/Th17 Balance	73, 85, 95
IFN‐γ	‐ T cells of colon tissue ‐ Sera	Decreased mRNA and protein expression	‐ Reducing inflammatory responses ‐ Inhibiting Th1/Th17 differentiation ‐ Improving Treg/Th17 Balance	73, 95
TNF‐α	‐ Colon tissue ‐ Macrophage ‐ Serum	Decreased protein expression	‐ Reducing inflammatory responses ‐ Inhibiting Th1/Th17 differentiation	73
IL‐10	‐ Colon tissue	Increased mRNA and protein expression	‐ Improving Treg/Th17 Balance ‐ Adjusting the M2/M1 ratio	73, 85, 95
IL‐22	‐ Colon tissue ‐ Serum	Increased protein expression	‐ Reducing inflammatory responses ‐ Inhibiting Th1/Th17 differentiation	73
*Autoimmune Encephalomyelitis*				
IL‐6	CD4^+^ T cells	Decreased protein expression	‐ Inhibiting Th1/Th17 differentiation	72
IL‐17				
IFN‐γ				
*Autoimmune Hepatitis*				
IL‐1β	‐ Hepatic tissue ‐ Serum	Decreased protein expression	‐ Reducing hepatic injury	208
IL‐2				
IFN‐γ				
TNF‐α				
IL‐10	‐ Hepatic tissue ‐ Serum	Increased protein expression	‐ Reducing hepatic injury	208
*Autoimmune Myocarditis*				
IL‐17	Serum	Decreased protein expression	‐ Inhibiting Th1/Th17 response ‐ Ameliorating autoimmune myocarditis	70
IFN‐γ				
*Autoimmune Uveoretinitis*				
IL‐1β	Dendritic cells	Decreased protein expression	‐ Inhibiting Th17‐ mediated autoimmune response	68
IL‐6				
IL‐23				

## 2 SUPPRESSIVE EFFECTS OF BERBERINE ON AUTOREACTIVE TH1/TH17 CELLS

Anomalous autoreactive responses of CD4^+^ T helper cells, such as Th1 cells producing IFNγ ^2^ and Th17 cells producing IL‐17,^47^, ^48^, ^49^, ^50^, ^51^, ^52^ are tightly correlated with the clinical severity and progression of several autoimmune diseases, clinically and experimentally.^11^, ^12^, ^53^, ^54^ Th1 and Th17 cells can also recruit other inflammatory cells into inflamed tissues through the secretion of several pro‐inflammatory cytokines, such as IFNγ, IL‐12, IL‐1β, IL‐2, IL‐17A, IL‐17F, IL‐21, IL‐22, IL‐23 and IL‐25 and GM‐CSF.^55^, ^56^ Th1 and Th17 cells differentiate from naïve CD4^+^ T cells through regulation by a complex network of transcription factors and cytokines. Janus kinase‐signal transducers and activators of transcription (JAK‐STAT) signalling is an important signalling transduction pathway regulating differentiation and function of Th1 and Th17 cells.^57^ The key members of the JAK/STAT family are STAT1 and STAT4 that, after IL‐12 stimulation, are activated by JAK2 and Tyrosine Kinase 2 (Tyk2) and participate in differentiation of Th1 cells.^58^, ^59^ On the other hand, STAT3 is an essential signalling mediator for the commitment of Th17 lineage and is provoked by TGF‐β, IL‐6 and IL‐23 cytokines (21‐23). The expression of these STAT‐signalling mediators is promoted by T‐bet transcription factor for STAT1 and STAT4 and RORγt transcription factor for STAT3.^30^, ^60^, ^61^

**FIGURE 1:**
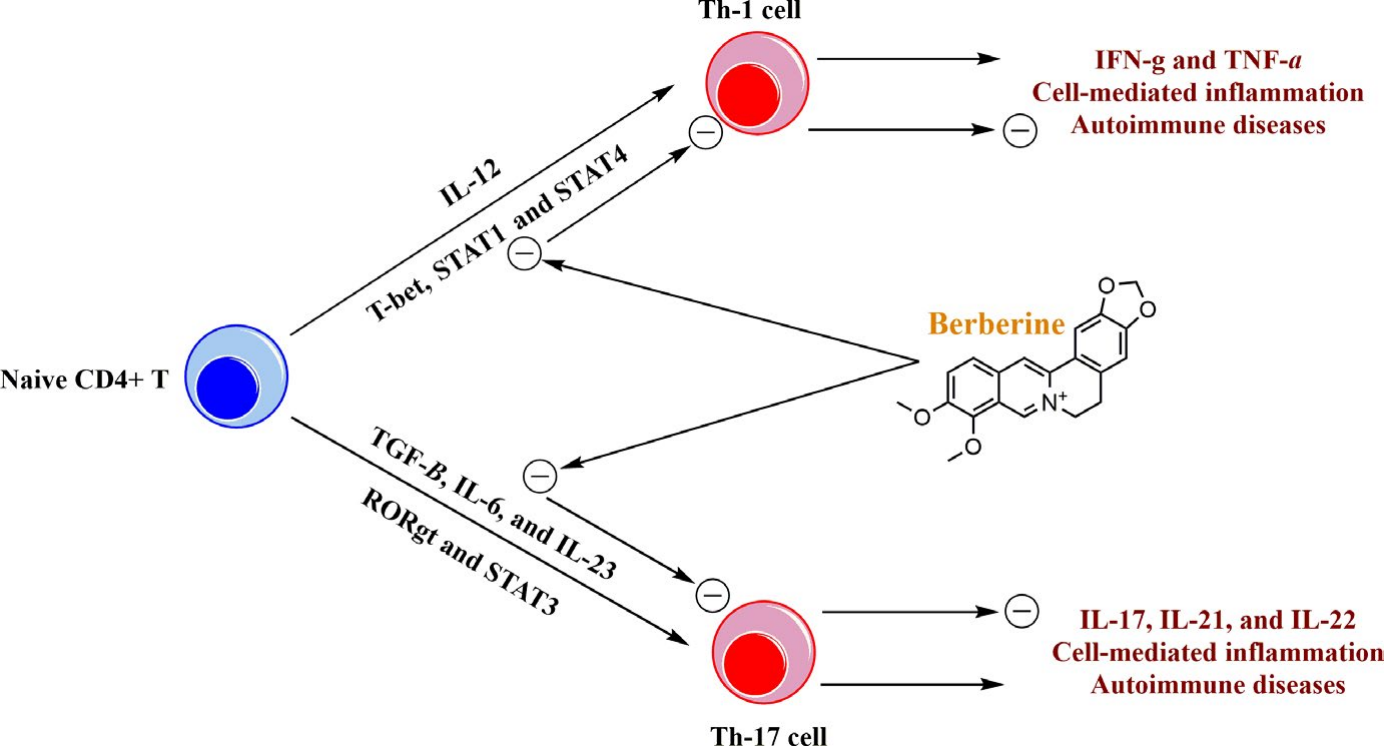
Suppressive effects of berberine on the differentiation and inflammatory function of Th‐1 and Th‐17 cells. Berberine can inhibit differentiation of naive CD4^+^ T cells into Th‐1 cells through down‐regulating the expression of IL‐12, STAT1, STAT4 and T‐bet, and into Th‐17 cells through down‐regulating the expression of TGF‐β, IL‐6, IL‐23, RORγt and STAT3. Suppressive effects of berberine on the differentiation of Th‐1 and Th‐17 cells leads to reduced levels of pro‐inflammatory cytokines and decreased autoimmune inflammation

An early study on mice model of autoimmune tubulointerstitial nephritis showed that berberine could reduce increased levels of Th1 cells, which was associated with an improvement in renal function.^62^ Experimental autoimmune neuritis (EAN) is a model of human Guillain‐Barre syndrome characterized by infiltration of the peripheral nervous system by autoreactive T cells promoting demyelination and axon damage.^63^, ^64^, ^65^ Berberine treatment was shown to ameliorate EAN severity by inhibiting the proliferation of CD4^+^ T cells and down‐regulating Th1 (TNF‐α) cytokine.^66^ Results from an ex vivo study on human CD4^+^ T cells isolated from patients with ocular Behcet's disease ^67^ and Vogt‐Koyanagi‐Harada disease ^68^ indicate that berberine can suppress Th17 responses through reducing the frequency of IL‐17 producing CD4^+^ T cells and inhibiting IL‐17 production. It is further confirmed by an in vivo study on AIA rats that showed berberine administration could significantly reduce the blood levels of Th17 population and the serum levels of IL‐17, which was accompanied by decreased expression of IL‐17 in synovium and Th17 transcription factor RORγt in the spleen.^69^ The further experimental study revealed that berberine treatment significantly attenuated the excessive response of Th1/Th17 cells through reducing elevated levels of Th1/Th17 cells and their cytokines IL‐17/IFNγ in rats with EAM, which was along with marked reduction in the impaired cardiac function and the pathophysiological severity.^70^ An in vitro study on naïve T cells isolated from the spleen of AIA rats indicated that berberine treatment could significantly decrease differentiation and survival of Th17 cells, in a concentration‐dependent manner, through down‐regulating surface marker CD196 and RORγt transcription factor.^71^ In mice with EAE, as a model of MS, treatment with berberine ameliorated the encephalitogenic autoreactive T cells by suppressing differentiation of naive CD4^+^ T cells into Th1 and Th17 cells.^72^ Suppressive effects of berberine on Th1/Th17 differentiation has been further confirmed by the other study conducted on mice with experimentally induced colitis in which such inflammatory cells are involved in the progression and severity of the disease.^73^ Mechanistically, berberine can decrease phosphorylation of STAT3 and expression of RORγt transcription factor during the differentiation of Th17 cells and down‐regulate phosphorylation of STAT4 and STAT1 and expression of T‐bet in differentiating Th1 cells (Figure 1).^70^, ^71^, ^72^ In sum, berberine can directly inhibit differentiation and function of Th1/Th17 cells and thereby decrease inflammation‐mediated severity and progression of autoimmunity disease. As discussed in the following sections, berberine can also suppress inflammatory responses of T cells through indirect effect *via* affecting function of Treg cells, DCs, and macrophages.

## 3 BENEFICIAL MODULATORY EFFECTS OF BERBERINE ON TREG/TH17 BALANCE

Treg cells are anti‐inflammatory cells that secrete inhibitory cytokines including IL‐10, IL‐35 and TGF‐β and, thereby, suppress the function of inflammatory Th1/Th17 cells.^27^, ^74^ Treg cells are often functionally defective and indicate only mild expansion in autoimmune disorders and are far from reaching numbers that can counterbalance the inflammatory immune responses.^27^, ^28^, ^75^ Treg/Th17 imbalance is an important hallmark of autoimmune disorders. In a model of autoimmune uveitis, berberine treatment was found to regulate Treg/Th17 balance, which was associated with an alteration in the composition of the intestinal microbiota, including an increase in the gut levels of *Akkermansia* genera, *Oscillibacter,* as well as *Lachnospiraceae* and *Ruminococcaceae*, and a decrease in *Lactobacilli* bacterial.^76^ The genera Akkermansia is known to suppress the negative effects of IFNγ and thereby decrease Th17 responses.^77^ *Lachnospiraceae* and *Ruminococcaceae* are butyrate‐producing bacteria that can increase the Treg/Th17 ratio.^78^, ^79^ Butyrate, a physiologically abundant short‐chain fatty acid (SCFA), has been suggested to involve in the regulation of T‐cell differentiation through several mechanisms, including the induction of G‐protein‐coupled receptor (GPCR) signalling, inhibition of de novo fatty acid synthesis through the deactivation of acetyl‐CoA carboxylase 1 and epigenetic regulation through inhibition of histone deacetylase activity, which are known to limit Th17 cell differentiation and promote Treg development.^79^ Likewise, *Oscillibacter* is a known producer of pentanoate and capable of enhancing the differentiation of Treg cells.^79^, ^80^ Pentanoate, another SCFA, through mechanisms similar to butyrate can promote IL‐10 production on Treg cells and inhibit Th17 generation.^81^ Moreover, *Lactobacillaceae*, a family of lactic acid bacteria, were shown to cause an increased type I IFN gene expression in the spleen and to worsen autoimmune manifestations.^82^, ^83^, ^84^

Modulation of Treg/Th17 responses by berberine through gut microbiota‐dependent regulation is also further supported in a model of ulcerative colitis in which berberine treatment decreased levels of gut bacteria including *Bacteroides*.^85^ *Bacteroides* are known to produce metabolites that induce Treg to produce IL‐10. Bacteroides have been reported to protect against experimental colitis through the release of polysaccharide A. This anti‐inflammatory effect is mediated by a decreased production of IL‐17 in the intestine and through the promotion of CD4^+^ T‐cell differentiation to IL‐10‐producing Treg.^86^, ^87^ However, mechanisms underlying the effects of berberine on the gut microbiota remain unclear yet and are needed to be taken into account in forthcoming studies.

**FIGURE 2:**
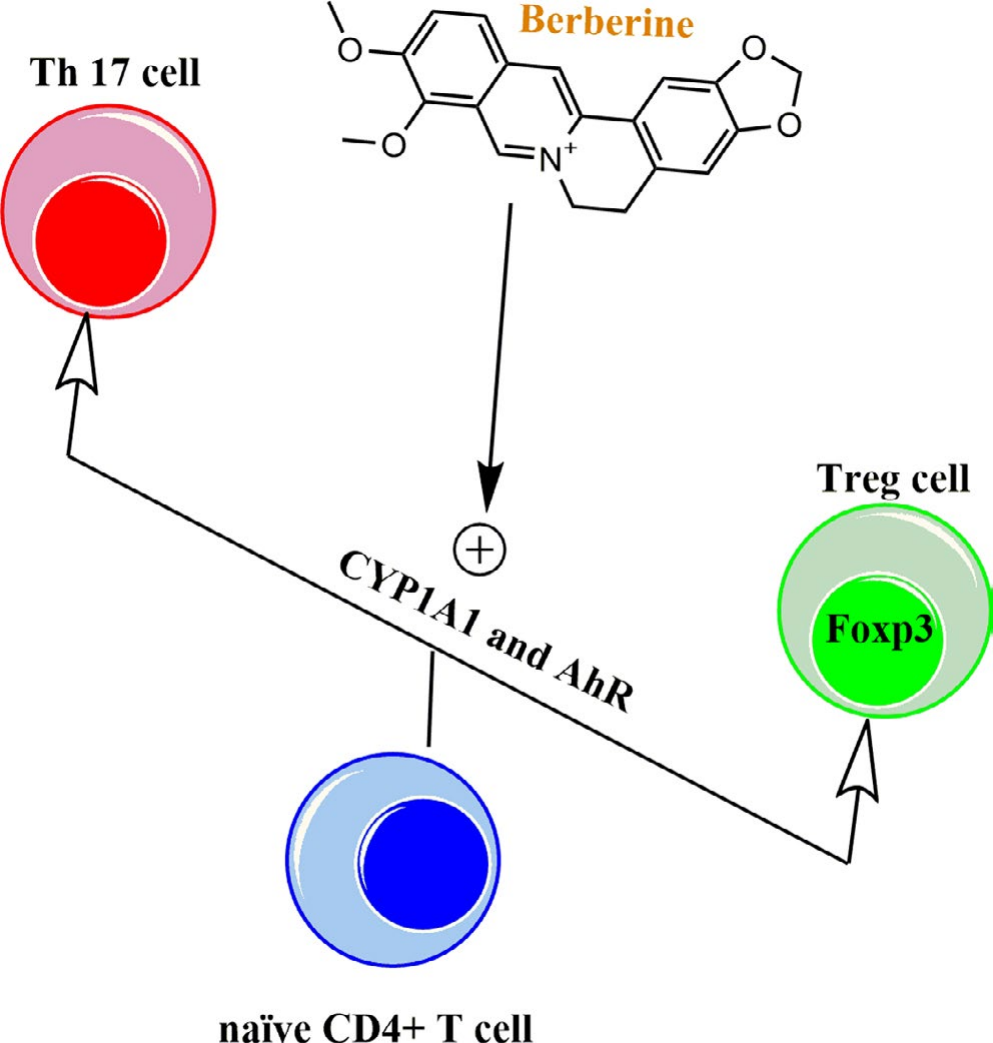
Mechanism underlying improving effects of berberine on Treg/Th17 balance. Berberine can shift differentiation of naïve CD4^+^ T cells into CD4^+^ Foxp3^+^ Treg cells through activating AhR transcription factor and up‐regulating CYP1A1

Improving effects of berberine on Treg/Th17 balance has been also confirmed in the other study that showed berberine could modulate differentiation of splenic naïve T cells of AIA rats; berberine treatment could shift differentiation of naïve CD4^+^ T cells into CD4^+^ Foxp3^+^ Treg cells, instead Th17 cells, through activating AhR/CYP1A1/Foxp3 axis.^88^ The differentiation and survival of Treg cells rely on the expression of Foxp3, which is induced by aryl hydrocarbon receptor (AhR) transcription factor and elevation in levels of cytochrome P450, family 1, subfamily A, polypeptide 1 (CYP1A1); a downstream element of AhR.^71^ In mechanism, berberine activates AhR transcription factor by which up‐regulates CYP1A1 levels and subsequently increases Foxp3 expression, leading to the differentiation of Treg cells (Figure 2).^88^ These findings can be further supported by reports that show berberine treatment can modulate Th17/Treg responses in other autoimmune diseases, such as colitis,^73^, ^85^, ^89^ type 1 diabetes,^90^ as well as EAE ^72^ and myocarditis.^70^

Taken together these findings suggest that berberine can beneficially modulate Treg/Th17 balance in autoimmune conditions, through two distinct mechanisms, directly by modulation of naïve CD4^+^ T cells’ differentiation and indirectly by affecting pattern of the gut microbiota.

## 4 MODULATORY EFFECTS OF BERBERINE ON DCS

The important players in T cell autoreactive responses are antigen‐presenting cells (APCs) like DCs, which provoke the complete activation of T cells by antigen presentation through the tri‐complex of antigen, MHC and TCR and exposing co‐stimulatory molecules, such as CD80 and CD86.^91^ These co‐stimulatory molecules interact with cognate ligands on Th1 and Th17 cells to derive a positive signal that is needed for T‐cell activation.^47^, ^92^ Moreover, APCs, particularly DCs, secrete pro‐inflammatory cytokines TNF‐α and IL‐12 or IL‐6 and TGF‐β that through activating the STAT signalling can induce differentiation of T cell lineage and thereby promote the generation of Th1 or Th17 cells, respectively.^93^, ^94^ There is evidence that shows the modulatory effects of berberine on T cells are, in part, APC‐dependent. Berberine was found to hamper APC function through reducing expression of co‐stimulatory molecules CD80 and CD86 as well as down‐regulating production of IL‐6 and IL‐12 cytokines.^72^, ^91^ In EAE mice, the modulatory effect of berberine on Th1 and Th17 cells was shown to be dependent on the inhibitory impact on APC‐derived IL12 and IL‐6, respectively.^72^ Another study on mice with IBD indicated that berberine reduced the production of TNF‐α, IL‐12, IL‐6 and TGF‐β in the maturated DCs and thereby decreased the population of Th1/Th17 cells in the mesenteric lymph nodes (MLNs), resulting in the amelioration of colon inflammation in colitis‐induced mice.^95^ In mechanism, berberine was shown to act as an antagonist at dopamine D1‐ and D2‐like receptors and whereby modulate cytokine production in DCs.^95^ Dopamine functions both as a hormone and a neurotransmitter, and there is evidence that dopamine involves in IBD development.^95^, ^96^, ^97^ During the development of IBD, the condition of the colon impairs the intracellular storage of dopamine in enteroendocrine cells and the enteric nervous system, which affects the associated inflammatory process.^96^ As for genetic evidence, the frequency of dopamine D2 receptor polymorphisms in IBD was similar in different groups of disease localization, behaviour and age of disease onset, supporting the involvement of dopamine receptors in IBD.^97^ Dopamine receptor subtypes are also known to be expressed on the surface of immune cells, such as DCs, where dopamine can bind during the development of IBD.^95^ Further supporting the involvement of dopamine receptor‐mediated signalling within immune cells in the progression of IBD is evidence that antagonists targeting dopamine D1‐like receptor and/or dopamine D2‐like receptor can prevent lipopolysaccharide (LPS)‐induced inflammation in lymphocytes and modulate cytokine secretion of DCs.^95^

Furthermore, the timely elimination of mature DCs is important to prevent aberrant activation of the inflammatory immune responses. Apoptosis deficiency in DCs leads to the accumulation and prolonged activity of DCs that, in turn, result in long‐last activation of lymphocytes and progression of autoimmunity responses.^98^ Berberine has been shown to exert anti‐apoptotic effects on DCs in in vitro and in vivo models of RA.^99^ Berberine could time‐ and dose‐dependently induce apoptosis in murine bone marrow(BM)‐derived DCs.^99^ Freshly isolated BM cells were found to be insensitive to berberine, and the susceptibility to berberine‐promoted apoptosis was increased during DC differentiation, in which mature IL‐12‐producing DCs showed higher sensitivity to berberine than immature DCs. Thus, berberine can selectively trigger apoptosis in mature DCs and whereby restrict DC maturation and shorten their lifespan.^99^ As mature DCs play the crucial roles in pathogenic inflammation and immune responses in autoimmune diseases, berberine‐induced apoptosis in mature DCs provides a major mechanism of immunomodulation that can be accounted, at least in part, for its immunosuppressive impacts observed in animal models of autoimmune diseases. Although the exact intracellular mechanisms underlying selective pro‐apoptotic effect in DCs remain unknown, it has been shown that the production of reactive oxygen species (ROS) and mitochondrial depolarization, as well as caspase 3 activation, are involved in berberine‐mediated apoptosis induction.^99^ In accordance with the aforementioned in vitro findings, berberine was indicated to markedly reduce the ratio of mature to immature DCs in spleens, confirming its selective pro‐apoptotic effect in mature DCs in vivo.^99^ In this regard, berberine treatment could cause a considerable loss of DCs and an elevation in the apoptosis of DCs within spleens and lymph nodes in AIA mice, which was accompanied by the antiarthritic and immunosuppressive effects in these mice.^99^

In sum, berberine has the potential to decrease survival and inflammatory functions of autoreactive APCs, mainly DCs, through inducing apoptosis and inhibiting co‐stimulatory molecules and inflammatory cytokine secretion, which is accompanied with reducing Th1/Th17 population and ameliorating severity and progression of autoimmune disorders.

## 5 MODULATORY EFFECTS OF BERBERINE ON INFLAMMATORY MACROPHAGES

Macrophages are the major innate immune cells present in almost every tissue or organ system and mainly act as phagocytic cells that engulf and digest cellular debris resulted from apoptosis, foreign substances, microbes and pathogens.^100^ In addition to acting as professional phagocytic cells, macrophages produce various cytokines, chemokines and growth factors whereby exhibit broad immunomodulatory, inflammatory and tissue‐repairing capabilities and actively promote the development of several autoimmune diseases.^100^, ^101^ For example, abnormally activated intestinal macrophages in IBD patients and experimental models of colitis produce various inflammatory cytokines, such as IFNγ, IL‐1β, IL‐6, IL‐17, IL‐23 and TNF‐α necessary for T‐cell differentiation, specifically inducing the production of Th1 and Th17 cells (191‐194). In berberine‐administrated mice with colitis, levels of such pro‐inflammatory cytokines in the colon and sera were significantly decreased, which was accompanied by a reduction in colonic macrophages and percentages of IL‐6^+^, IL‐1β^+^ and TNF‐α^+^ secreting macrophages among splenocytes.^73^, ^102^ Moreover, macrophage infiltration in the inflamed lesions is one of the most important hallmarks of many autoimmune disorders such as IBD,^103^, ^104^ and berberine was shown to decrease macrophage infiltration into the colon by promoting apoptosis and inhibit signalling pathways involved in the stimulation of pro‐inflammatory cytokine production, including MAPK and NF‐κB, in colonic macrophages of mice with colitis.^102^ Macrophages differentiate into two different polarization states serving opposite functions: classical M1 phenotype, which generates pro‐inflammatory cytokines; participate as inducer and effector cells in polarized Th1 responses; derive resistance against intracellular pathogens and tumours; and promote tissue destruction, and alternative M2 subsets, which generate anti‐inflammatory cytokines and contribute to tissue repair and remodelling as well as tumour progression.^105^, ^106^ Of note, it was indicated that anti‐inflammatory M2 macrophages become the more dominant macrophage population after berberine treatment in colitis mice.^73^ In conclusion, the inhibitory effect of berberine on inflammatory macrophages can be considered as another mechanism through which ameliorates autoreactive T‐cell responses in autoimmune disorders.

## 6 BERBERINE‐MEDIATED ATTENUATING DEMYELINATION AND AUTOIMMUNE INFLAMMATION IN THE CENTRAL NERVOUS SYSTEM

As berberine can cross the blood‐brain barrier (BBB), evaluating the beneficial effects of berberine on neurodegenerative diseases has attracted extensive attention.^107^, ^108^ Berberine treatment has been indicated to effectively ameliorate the severity of EAE in C57 BL/6 mice either when evaluated clinically or by neuropathological criteria. Berberine significantly decreased the severity of clinical symptoms including the loss of tail tonicity, flaccid tail, ataxia and/or paresis of hindlimbs, complete paralysis of hindlimbs, as well as moribund or death in EAE mice. Neuropathological manifestations including the demyelination in the lumbar spinal cords and infiltration of inflammatory cells such as macrophages, T and B lymphocytes into CNS white matter in the lumbar spinal cords were markedly alleviated in berberine‐treated EAE mice.^109^, ^110^ Elevated permeability of BBB is mainly responsible for the infiltration of leucocytes into CNS and plays a key role in the initiation and progression of MS and EAE,^111^ whereas preventing BBB alterations limits the severity and progression of the disease.^112^, ^113^ Interestingly, berberine‐mediated reduction of leucocyte infiltration and CNS inflammation in treated EAE mice was indicated to be due to reduced BBE permeability.^109^ Of note, BBB permeability is known to be elevated by MMPs,^114^, ^115^ particularly the gelatinases, MMP‐2 and MMP‐9,^115^, ^116^ which exist in the brain and the cerebrospinal fluid (CSF) and govern migration of cells across the BBB through degrading type IV collagen and disrupting other components of the extracellular matrix surrounding blood vessels, resulting in disruption of the BBB integrity.^117^, ^118^, ^119^ Inhibitory effect of berberine on BBB permeability was shown to be in part due to reducing the activity and expression of MMP‐9 in the brain and CSF of treated EAE mice.^109^, ^110^ MMPs also degrade laminins that are the major components of the extracellular matrix participating in neuronal development, survival, and regeneration.^120^ Matrix proteins such as laminins are also widely disseminated throughout the brain parenchyma, and loss of parenchymal laminins may affect cell‐matrix interactions and cell survival.^121^, ^122^, ^123^, ^124^ The destruction of laminins around nerve cells by MMP‐9 can disrupt cell‐matrix interactions and further contribute to neuronal cell death.^125^, ^126^ Berberine administration could exert a neuroprotective effect on the brain following EAE through up‐regulating laminin activity simultaneously accompanied with the diminished MMP‐9 activity, which resulted in the decreased neuronal apoptosis.^127^

In response to CNS pathologies, astrocytes are activated and the degree of their reactivity positively associates with the severity of MS and EAE.^128^, ^129^ Sphingosine‐1‐phosphate (S1P) is a lipid that binds to S1P1 in astrocytes and promotes essential steps in the pathogenesis of EAE through inducing the release of interleukins and other cytokines that mediate inflammatory responses.^130^ Sphingosine kinase 1 (SphK1) is a kinase that phosphorylates and activates S1P,^131^ and up‐regulated SphK1/S1P signalling is one of the key factors involves in astrocytes‐mediated inflammatory responses in MS pathogenesis.^131^, ^132^ Mechanistical study indicates that berberine can decrease demyelination and loss of neurophysiological function in EAE mice by suppressing the SphK1/S1P signalling pathway in astrocytes.^110^ To sum up, berberine can attenuate clinical and pathological parameters of EAE in mice through reducing the demyelination in the lumbar spinal cords and alleviating leucocyte infiltration and CNS inflammation, together with neuroprotective effect, by maintaining BBB integrity and increasing parenchymal laminins *via* inhibiting MMP‐9 and SphK1/S1P signalling in the CSF and brain.

## 7 BERBERINE‐MEDIATED ATTENUATING AUTOIMMUNE INFLAMMATION IN THE PERIPHERAL NERVOUS SYSTEM

Guillain‐Barre syndrome (GBS) is an autoimmune disease attacking the peripheral nervous system (PNS) and characterized by inflammatory demyelination and axon damage. Experimental autoimmune neuritis (EAN) is a commonly‐used animal model recapitulating clinical symptoms and pathological features of human GBS ^63^ which is promoted by immunization with PNS myelin proteins or corresponding neurogenic peptides, such as P0 peptide 180‐199, combined with Freund's complete adjuvant.^64^, ^133^

The hallmark of EAN is PNS infiltration by inflammatory cells, particularly Th1 cells and macrophages, which secrete pro‐inflammatory cytokines such as TNF‐α at local sites of inflammation. Interestingly, berberine treatment was shown to significantly ameliorate EAN by suppressing both cellular and humoural immunity that are implicated in GBS/EAN.^66^ In berberine‐treated EAN rats, clinical symptoms, including flaccid or dragging tail and hind limb paraparesis, were detected to be significantly alleviated.^66^ The ameliorating effect of berberine on EAN was found to be accompanied by an inhibited proliferation of CD4^+^ T cells, down‐regulated both Th1 (TNF‐α) and Th2 (IL‐10) cytokines and decreased anti‐P0 peptide IgG1 and IgG2a.^66^

Pro‐inflammatory cytokines, such as TNF‐α, have a destructive role in several autoimmune diseases, such as GBS,^134^, ^135^, ^136^ Crohn's disease,^137^ RA ^138^ and MS.^139^ TNF‐α further promotes and recruits inflammatory cells ^140^ and decreases the permeability of the blood‐nerve barrier (BNB), through inducing MMPs to facilitate the infiltration.^141^ In addition, TNF‐α suppresses the proliferation of Schwann cells (SC) and promotes SC death.^142^, ^143^ Therefore, the ameliorating effect of berberine on clinical symptoms of EAN can, in part, stem from inhibitory effects on TNF‐α secretion by Th1 cells.^66^ Mitogen‐activated protein kinase (MAPK) signalling, a regulator of TNF‐α production, is known to be suppressed by berberine and can be a possible mechanism for the inhibitory effect of berberine on TNF‐α secretion by Th1 cells in EAN.^66^

Besides, growing evidence shows that IL‐10, commonly known as an anti‐inflammatory cytokine, plays a key role in both the initiation and progression of autoimmune diseases, through activating proliferation and antibody production of B cells.^144^, ^145^, ^146^ In GBS and EAN, IL‐10 secretion is elevated and positively associated with axonal nerve damage and antiganglioside antibodies.^146^ This can explain the protective effects of berberine against neuropathy in EAN mice; however, underlying mechanisms remain largely unknown.^66^

## 8 AMELIORATING EFFECTS OF BERBERINE ON OCULAR MANIFESTATIONS AND AUTOIMMUNE INFLAMMATION OF UVEITIS

Uveitis is a blinding intraocular inflammatory disorder caused by an autoimmune response implicated the uveal layers, the retina and vitreous.^147^, ^148^ Autoreactive retina‐specific T cells that secrete IFN‐γ or IL‐17A are generated in lymph nodes and spleen and cross the blood‐retinal barrier (BRB), whereafter inflammatory cells are recruited into the retina that eventually leads to full‐blown uveoretinal inflammation.^149^, ^150^ EAU is a widely used model of autoimmune uveitis in humans, possessing an acute and severe inflammation involving both the anterior and posterior segments of the eye.^151^, ^152^

Of note, berberine has been found to ameliorate ocular manifestations of EAU in the experimental model.^76^, ^153^ Berberine‐treated EAU mice showed alleviated anterior chamber inflammation and attenuated clinical manifestations including corneal oedema, ciliary injection of the cornea, and cells in the aqueous humour as well as posterior synechiae.^76^ Berberine treatment could also attenuate BRB breakdown and decrease histological characteristics of uveitis, including massive inflammatory cells in the choroid and retina, retinal folds and damage of photoreceptor cells.^76^ Such inhibitory effect of berberine on ocular manifestations of EAU was found to be accompanied by a decreased frequency of pathogenic Th1 and Th17 cells and a small elevation of Tregs as well as a remarkable alteration in intestinal microbial composition (as discussed with detail in the previous section).^76^ Similar results were also reported by another study on berberine‐treated EAU rats.^153^ These findings are further supported by clinical studies showing blocking effects of berberine on inflammatory T cells in patients with Ocular Behcet's disease ^67^ and/or Vogt‐Koyanagi‐Harada,^68^ the most common causes of uveitis.

## 9 CONCLUSION

**FIGURE 3:**
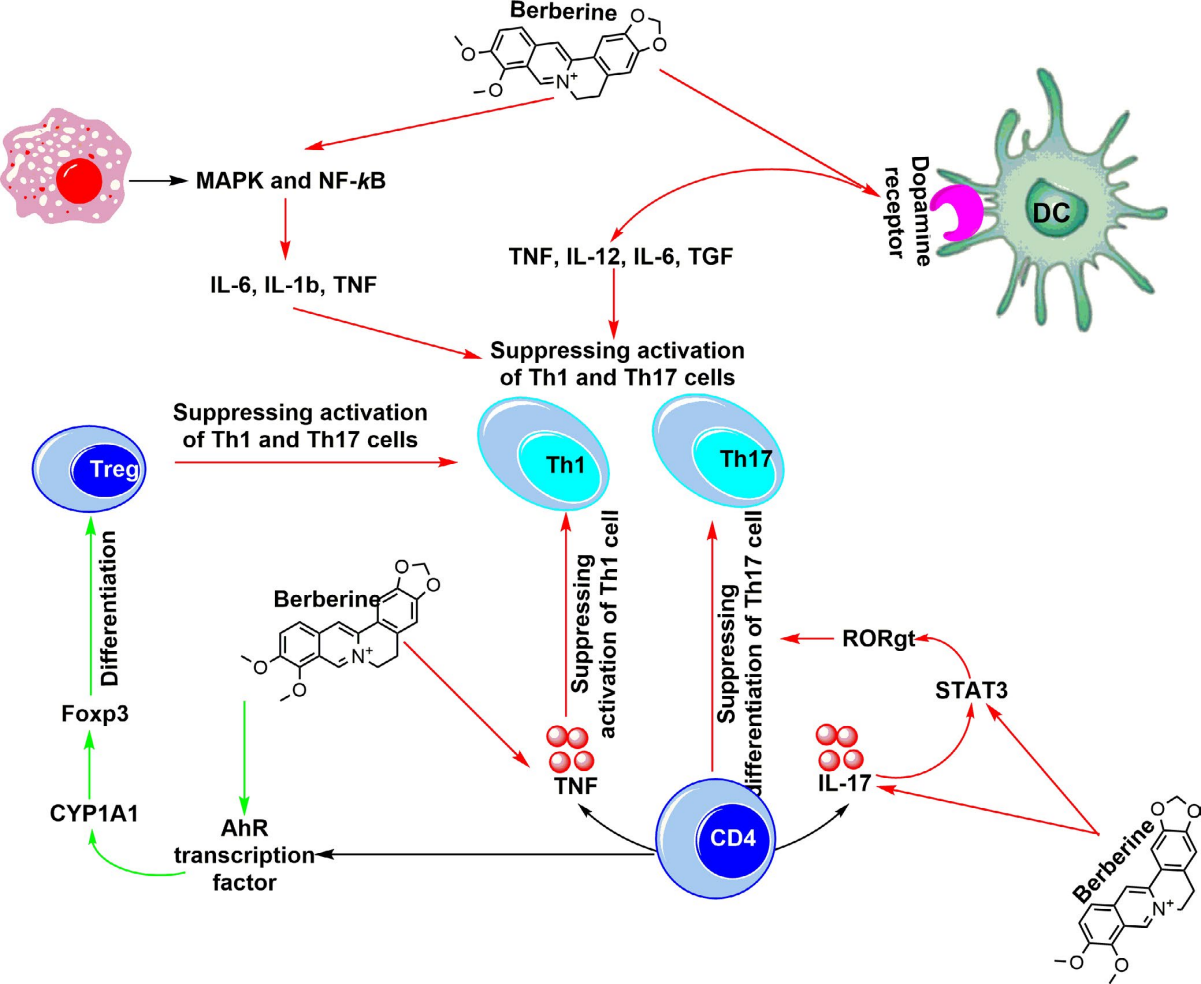
The direct and indirect inhibitory effects of berberine on autoreactive Th17 and Th1 cells. Through the direct route, berberine suppresses differentiation of CD4 cells into Th17 and Th1 cells and inhibits activation of these cells through decreasing expression of TNF and IL17 cytokines via inhibiting STAT3 and RORgt. Through the indirect route, berberine suppresses the activation of both Th17 and Th1 cells via modulating the activity of macrophages and DCs via suppressing the production of inflammatory cytokine. Further suppressive effect of berberine on Th17 and Th1 cells is achieved through its promoting impact on Treg differentiation and activation via inducing activity of AhR transcription factor, CYP1A1 and Foxp3. Green and red arrows reflect promoting and inhibiting effects of berberine, respectively

Growing evidence witnessed by the in vitro and in vivo experimental studies reveals that berberine has the potential to ameliorate destructive autoreactive inflammation in autoimmune conditions (Figure 3). Berberine can directly suppress pro‐inflammatory responses of Th1 and Th17 cells by inhibiting the function and differentiation of these cells, mechanistically, through hampering STAT and RORγt signalling pathways. Berberine is also found to indirectly decrease Th cell‐mediated inflammation through modulating or suppressing other cells assisting autoreactive inflammation, such as Tregs, DCs and macrophages. Imbalance of Treg/Th17 cells is an important hallmark of autoimmune disorders, and berberine has been found to induce differentiation of Tregs in autoimmune conditions through two distinct mechanisms, directly by modulation of naïve CD4^+^ T cells’ differentiation and indirectly by affecting pattern of the gut microbiota. Also, berberine can decrease survival and inflammatory functions of DCs through inducing apoptosis and inhibiting co‐stimulatory molecules and inflammatory cytokine secretion, which is accompanied with a reduction of Th1/Th17 population and amelioration of the severity and progression of autoimmune complications. Likewise, berberine treatment can elevate the population of anti‐inflammatory M2 macrophages and suppress M1 macrophages producing pro‐inflammatory cytokines, resulting in amelioration of autoreactive T‐cell responses in autoimmune disorders. To our knowledge, all reported ameliorating effects of berberine on T cell‐mediated autoimmune inflammation are based on preclinical and cell culture investigations. Hence, further investigations are required to determine the clinical efficiency of berberine in patients with autoimmunity.

## CONFLICT OF INTEREST

We wish to confirm that there are no known conflicts of interest associated with this publication and there has been no significant financial support for this work that could have influenced its outcome.

## AUTHOR CONTRIBUTIONS

**Seyed‐Morteza Ehteshamfar:** Writing‐original draft (equal). **Masoume Akhbari:** Writing‐review & editing (lead). **Jalil Tavakol Afshari:** Validation (lead). **Motahareh Seyedi:** Software (lead). **Banafsheh Nikfar:** Investigation (equal). **Abbas Shapouri‐Moghaddam:** Supervision (equal); Validation (equal). **Erfan Ghanbarzadeh:** Investigation (equal); Project administration (equal). **Amir Abaas Momtazi‐Borojeni:** Conceptualization (lead); Project administration (supporting); Supervision (equal); Validation (equal).

## Data Availability

Data sharing is not applicable to this article as no new data were created or analysed in this study.
